# Gene expression profiling of the olfactory tissues of sex-separated and sex-combined female and male mice

**DOI:** 10.1038/sdata.2018.260

**Published:** 2018-12-04

**Authors:** Stephen W. Santoro, Susanne Jakob

**Affiliations:** 1 Neuroscience Program, Dept. of Zoology & Physiology, University of Wyoming, Laramie, WY, USA; 2 Department of Stem Cell and Regenerative Biology, Harvard University, Cambridge, MA, USA

**Keywords:** Olfactory receptors, Sexual dimorphism, Transcriptomics

## Abstract

Olfactory experience can alter the molecular and cellular composition of chemosensory neurons within the olfactory sensory epithelia of mice. We sought to investigate the scope of cellular and molecular changes within a mouse’s olfactory system as a function of its exposure to complex and salient sets of odors: those emitted from members of the opposite sex. We housed mice either separated from members of the opposite sex (sex-separated) or together with members of the opposite sex (sex-combined) until six months of age, resulting in the generation of four cohorts of mice. From each mouse, the main olfactory epithelium (MOE), vomeronasal organ (VNO), and olfactory bulb (OB) were removed and RNA-extracted. A total of 36 RNA samples, representing three biological replicates per sex/condition/tissue combination, were analyzed for integrity and used to prepare RNA-seq libraries, which were subsequently analyzed via qPCR for the presence of tissue- or sex-specific markers. Libraries were paired-end sequenced to a depth of ~20 million fragments per replicate and the data were analyzed using the Tuxedo suite.

## Background & Summary

Sensory activity plays an important role in guiding the development of the nervous system, in part through activity-dependent changes in gene expression^1–3^. In the olfactory system, activity mediates the formation and the refinement of connections between olfactory sensory neurons (OSNs) located in the MOE and postsynaptic neurons in the OB^4–8^, as well as the relative abundance of OSNs that express specific olfactory receptor (OR) genes^9–15^. The latter changes appear to occur *via* alterations in the turnover rates of specific OSNs, which are continually born and replaced throughout life^16,17^. Like OSNs, Vomeronasal sensory neurons (VSNs) also undergo turnover throughout life^17^, suggesting that the abundance of VSN subtypes may have a similar capacity for experience-dependent changes. Activity-dependent changes to the representation of chemosensory neurons have been hypothesized to play a role in adapting an individual’s olfactory system to the detection and/or discrimination of salient odors, which may vary from one olfactory environment to another^11^.

The datasets described here were generated to enable investigation of the scope of molecular and cellular changes that occur within the olfactory system as a function of mouse exposure to odors from the opposite sex for a prolonged time period. Mouse odors are complex mixtures of volatile and non-volatile chemicals derived from skin secretions and substances such as urine, tears, saliva, and feces that are known to differ substantially between males and females^18–27^ and activate distinct subsets of OSNs and VSNs^18,20,21,28–35^. Because male and female mice emit distinct odor profiles, we predicted that sex-separated males and females would have distinct olfactory experiences and would thus display differences in their profiles of olfactory sensory neuron subtypes and gene expression. In contrast, sex-combined male and female mice would be expected to have more similar olfactory experiences and would thus display fewer differences in their profiles of OSN/VSN subtypes and gene expression.

**Figure 1:**
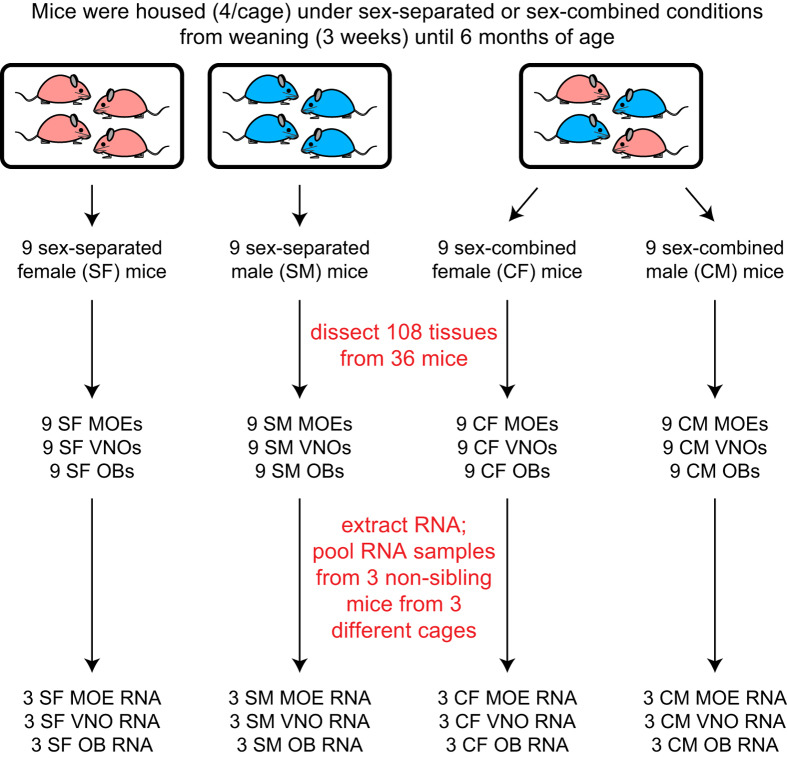
Experimental design. From weaning (P21) until 6 months of age, mice experienced either a sex-separated environment, in which they were housed either 4 females/cage (SF mice; *left*) or 4 males/cage (SM mice; *middle*), or a sex-combined environment (CF and CM mice; *right*), in which they were housed 2 females + 2 males/cage. MOE, VNO, and OB tissues were dissected from each of 9 mice per sex/condition combination, resulting in a total of 108 tissue samples. RNA was extracted from each tissue sample and pooled in groups of 3, resulting in 36 RNA samples (3 biological replicates per sex/condition/tissue combination), and used to generate RNA-seq libraries.

To generate the datasets described here, we housed male and female mice either separated from members of the opposite sex (sex-separated) or combined with members of the opposite sex (sex-combined) from the time of weaning until six months of age (Fig. 1). We then dissected the MOE, VNO, and OB tissues from a total of 36 mice (six mice per sex/condition/tissue combination) and extracted the RNA from each tissue. We generated a total of 108 RNA samples, which were combined in groups of 3 to generate a total of 36 pooled-RNA samples, with each sex/condition/tissue combination represented by three biological replicates. The integrity of each of the 36 pooled-RNA samples was analyzed and each sample was used for the generation of a stranded RNA-seq library. Libraries were analyzed by quantitative PCR (qPCR) for the presence or absence of tissue- and sex-specific markers and then paired-end sequenced to generate a total of approximately 20 million sequence pairs per library. Sequences were aligned to the mouse genome and gene expression was quantified using the Tuxedo suite^36^. Further analyses of the data, including assessment of the effects of sex separation on chemosensory neuron abundance and overall gene expression, have been published in a separate manuscript^37^.

## Methods

These methods represent an expanded version of some of the methods described in our related work^37^. All procedures involving animals were carried out in accordance with NIH standards and approved by the University of Wyoming and Harvard University Institutional Animal Care and Use Committees (IACUC).

### Preparation of olfactory tissues from sex-separated and sex-combined mice

C57Bl/6 mice were subjected to either sex-separated (SF and SM samples) or sex-combined (CF and CM samples) conditions, in which animals were housed four females per cage (SF), four males per cage (SM), or two females and two males per cage (CF and CM) from weaning (postnatal day 21) until 6 months of age (Fig. 1). At the time of weaning, SF and SM cages were transferred to rooms containing only mice of the same sex to avoid exposure to opposite-sex odors from cages in the same room. Pups born in the sex-combined cages were euthanized within one day of birth to minimize exposure to pup odors. At 6 months of age, mice were sacrificed and MOE, VNO, and OB tissues were dissected as described^11^. Briefly, dissections were performed as follows: Using strong scissors, mice were decapitated and the bottom jaw was removed, along with and the skin, soft tissue, front teeth, and palate. Using fine scissors, the cranium was cut down the midline above the brain from the brainstem to the OB. Following removal of the dorsal and lateral cranial bones, including the bones surrounding the OB, the brain and OB were carefully lifted from the cranium and the OB was separated from the brain with a scalpel. The whole VNO was obtained by breaking the vomer bone with forceps and carefully lifting the vomer bone and attached VNO from the ventral nasal cavity. Finally the MOE was obtained by removing the dorsal and lateral bones surrounding the MOE and carefully lifting it from the dorsal nasal cavity. Immediately following dissection, each tissue was placed in a sterile microcentrifuge tube, flash-frozen on dry ice, and stored at −80 °C until RNA extraction.

### RNA-seq analysis

**Figure 2:**
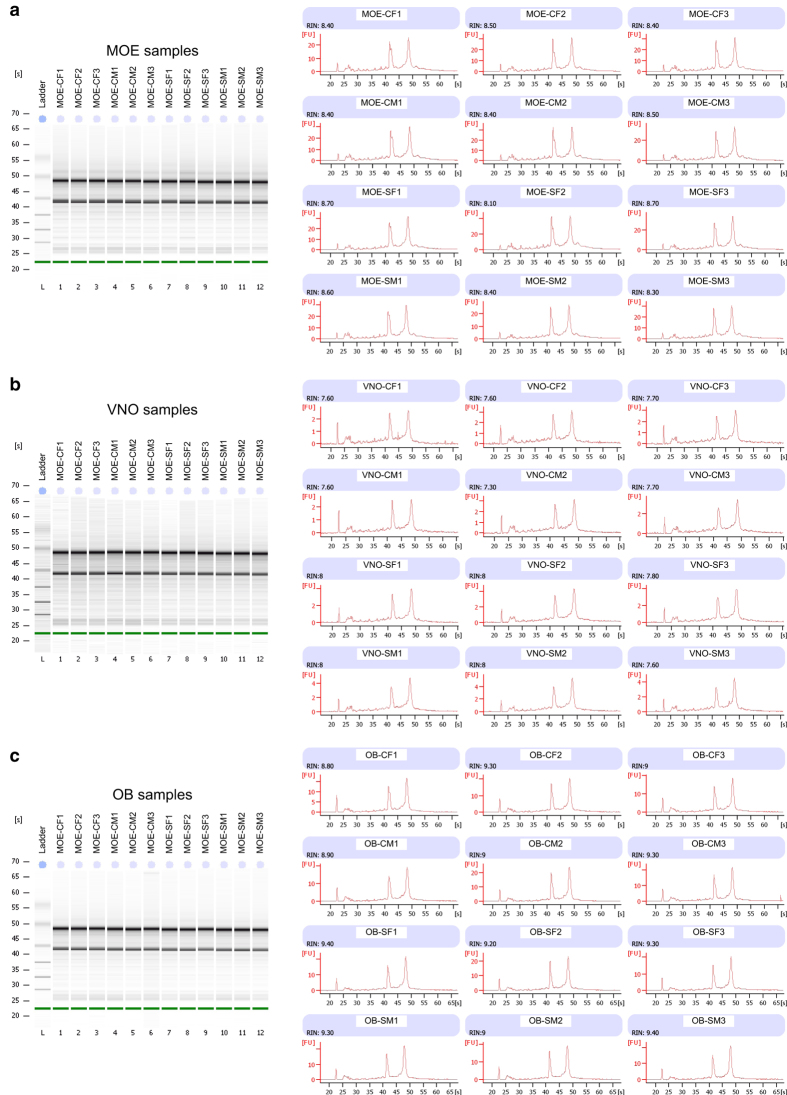
RNA integrity analysis. The integrity of (**a**) MOE, (**b**) VNO, and (**c**) OB samples was analyzed using an Agilent Bioanalyzer 2100 instrument. RNA integrity number (RIN) values for all samples are listed in Table 1.

For each combination of tissue (MOE, VNO, OB), sex (F, M), and condition (sex-separated [S], sex-combined [C]), six individual RNA samples were prepared from six individual tissue samples (from 6 individual mice) *via* mechanical homogenization in Trizol Reagent (Life Technologies) following the manufacturer’s protocol, resulting in a total of 108 RNA samples (Fig. 1). Trizol-purified RNA samples were quantified using a NanoDrop instrument (ThermoFisher Scientific). Equal quantities of three samples of the same sex/condition/tissue were combined and further purified using an RNeasy Plus Mini Kit (Qiagen) to generate 36 samples, representing three biological replicates per combination of sex/condition/tissue. Integrity of the RNA was analyzed using a 2100 Bioanalyzer (Agilent) (Fig. 2; Table 1).

**Figure 3:**
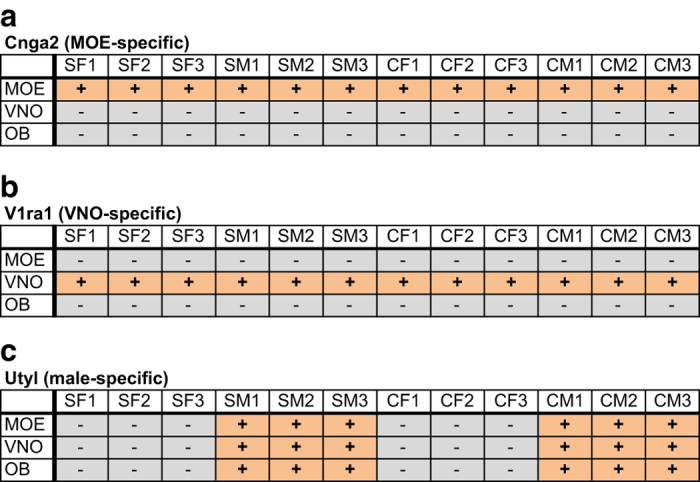
qPCR validation of RNA-seq libraries. Libraries were analyzed by qPCR for the presence (+) or absence (−) of (**a**) *Cnga2*, a gene expressed in MOE but not VNO or OB tissues, (**b**) *Vmn1r51*, a gene expressed in VNO but not OB or MOE tissues, and (**c**) *Utyl*, a gene expressed in male but not female tissues.

**Figure 4:**
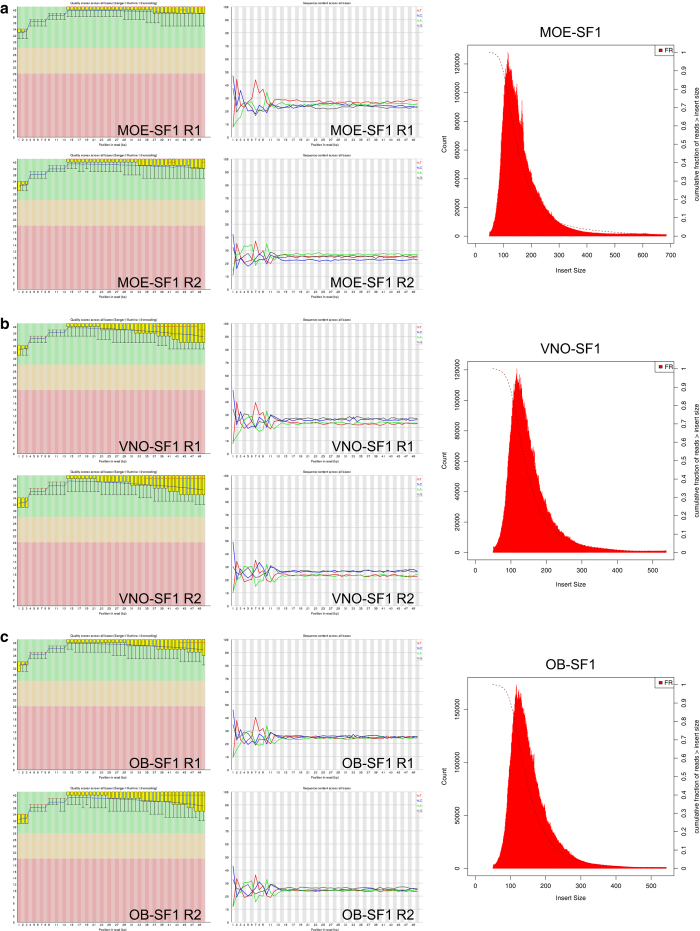
Analysis of library sequence quality and insert lengths. (*Left*) Per base quality and (*middle*) per base sequence analyses from FASTQC (Andrews S. (2010). FastQC: a quality control tool for high throughput sequence data. Available online at: http://www.bioinformatics.babraham.ac.uk/projects/fastqc/). (*Right*) Insert length analyses from the CollectInsertSizeMetrics tool (http://broadinstitute.github.io/picard/). Figures are for analysis of the MOE-SF1, VNO-SF1, OB-SF1 samples, which were representative.

**Figure 5:**
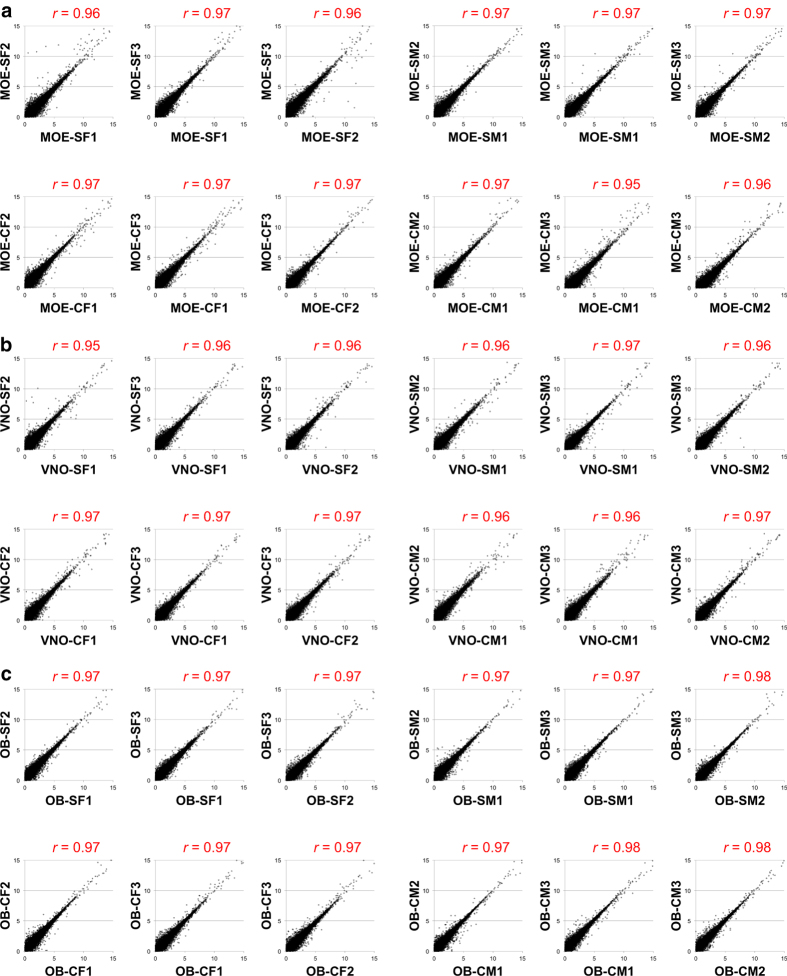
Analysis of the correlation of gene expression between biological replicates. Pairwise scatter plots of log2-transformed FPKM values and correlation coefficients for biological replicates of (**a**) MOE, (**b**) VNO, and (**c**) OB samples. Pearson correlation coefficients (*r*) for each comparison are indicated in red.

Using the TruSeq Stranded Total RNA with Ribo-Zero Gold Kit (Illumina), each RNA sample was depleted of ribosomal RNA and used to prepare an RNA-seq library tagged with a unique barcode. Library identity and quality were confirmed *via* quantitative PCR (qPCR) analysis using primers specific for genes expressed in the MOE (*Cnga2*: TCTGTTGGTAGCCAGAGCCT and AGCCCTTGTTCTAGGAAGCC), VNO (*Vmn1r51*: TGAGAACAGCAGGGTACACA and TGAATGCCATGACCAGTAGC), and male tissues (*Utyl*: GGTTCAGTGCACTTGCCTTT and TGATCCCTAGCTACTTGTCTGTTTT) (Fig. 3). Libraries were quantified using a Qubit instrument (ThermoFisher Scientific). Libraries were paired-end sequenced (2 × 50 bases) to a depth of ~40 million reads/sample (~20 million paired-end fragments/replicate; Table 2) using a HighSeq 2000 instrument (Illumina). Sequencing for each sample was split between two lanes, resulting in the generation of four FASTQ files (Lane 1 R1, Lane 1 R2, Lane 2 R1, and Lane 2 R2) per sample. FASTQ files were analyzed for quality using FASTQC (Fig. 4; Andrews S. (2010). FastQC: a quality control tool for high throughput sequence data. Available online at: http://www.bioinformatics.babraham.ac.uk/projects/fastqc/). For each library, Read 1 (R1) FASTQ files from Lanes 1 and 2 were merged and Read 2 (R2) FASTQ files from Lanes 1 and 2 were merged, resulting in a single R1 FASTQ file and a single R2 FASTQ file for each library. Merged R1 and R2 FASTQ files were analyzed using the Tuxedo suite^36^ on the Galaxy platform (https://usegalaxy.org)^38^. For each sample, sequence pairs were aligned to the genome using Tophat2^39^, resulting in concordant alignments for ~80% of the read pairs (Table 2). Analyses of gene expression levels and differential expression were performed using Cufflinks and Cuffdiff, respectively^36^. The correlation of FPKM values between biological replicates was analyzed pairwise (Fig. 5). Significance testing for differential expression was performed on all genes with a minimum alignment count of 5 fragments.

## Data Records

RNA-seq data files in FASTQ format were deposited at NCBI Sequence Read Archive (Data Citation 1). This accession contains a total of 144 FASTQ files resulting from paired-end sequencing for each of the 36 samples on two lanes. The FASTQ data were used to generate FPKM values for each sample. The processed data were deposited at NCBI Gene Expression Omnibus (Data Citation 2).

## Technical Validation

### Validation of RNA samples

Following the extraction, pooling, and purification of RNA samples and prior to their use in RNA-seq library preparation, the integrity of all 36 samples was analyzed using an Agilent 2100 Bioanalyzer. This analysis revealed RNA integrity number (RIN) values of at least 8.3, 7.3, and 8.8 for each of the MOE, VNO, and OB samples, respectively (Fig. 2, Table 1). These values were deemed satisfactory for RNA-seq library preparation using the TruSeq Stranded Total RNA kit.

### Validation of RNA-seq libraries

Prior to sequencing, all libraries were analyzed by qPCR for the presence (or absence) of the following gene markers: *Cnga2*, a gene expressed in MOE but not VNO or OB tissues, *Vmn1r51 (V1ra1)*, a gene expressed in VNO but not OB or MOE tissues, and *Utyl*, a gene expressed in male but not female tissues. This analysis revealed that *Cnga2* expression was detected only in the MOE libraries, *Vmn1r51* expression was detected only in the VNO libraries, and *Utyl* expression was detected only in the male libraries (Fig. 3).

### Validation of sequencing data and alignments

FASTQ files obtained from Illumina sequencing were analyzed for quality using FASTQC (Andrews S. (2010). FastQC: a quality control tool for high throughput sequence data. Available online at: http://www.bioinformatics.babraham.ac.uk/projects/fastqc/). This analysis revealed that the raw sequence data was of high quality (Fig. 4). Alignment of the libraries resulted in an average of 90.1% of reads aligned to the mouse genome and 81.9% of pairs aligned concordantly (Table 2). Analysis of the sequenced libraries using the CollectInsertSizeMetrics tool (http://broadinstitute.github.io/picard/) revealed a mean insert size for all libraries of 167 bp. (Table 2; Fig. 4). Following Cufflinks determination of gene expression values (FPKM) for each gene in each library, the pairwise correlation of FPKM values between biological replicates were analyzed (Fig. 5). This analysis revealed mean Pearson correlation coefficients (*r*) of 0.97, 0.96, and 0.97 for the MOE, VNO, and OB replicates, respectively (Fig. 5).

## Additional information

**How to cite this article**: Santoro, S. W. *et al.* Gene expression profiling of the olfactory tissues of sex-separated and sex-combined female and male mice. *Sci. Data*. 5:180260 doi: 10.1038/sdata.2018.260 (2018).

**Publisher’s note**: Springer Nature remains neutral with regard to jurisdictional claims in published maps and institutional affiliations.

## Supplementary Material



## Figures and Tables

**Table 1:** RNA samples used for library preparation.

Sample ID	RIN	Description	Index	Index seq
MOE-SF1	8.7	MOE, 6m Female, sex separated	AR013	AGTCAA
MOE-SF2	8.1	MOE, 6m Female, sex separated	AR014	AGTTCC
MOE-SF3	8.7	MOE, 6m Female, sex separated	AR015	ATGTCA
MOE-SM1	8.6	MOE, 6m Male, sex separated	AR016	CCGTCC
MOE-SM2	8.4	MOE, 6m Male, sex separated	AR018	GTCCGC
MOE-SM3	8.3	MOE, 6m Male, sex separated	AR019	GTGAAA
MOE-CF1	8.4	MOE, 6m Female, sex combined	AR002	CGATGT
MOE-CF2	8.5	MOE, 6m Female, sex combined	AR004	TGACCA
MOE-CF3	8.4	MOE, 6m Female, sex combined	AR005	ACAGTG
MOE-CM1	8.4	MOE, 6m Male, sex combined	AR006	GCCAAT
MOE-CM2	8.4	MOE, 6m Male, sex combined	AR007	CAGATC
MOE-CM3	8.5	MOE, 6m Male, sex combined	AR012	CTTGTA
VNO-SF1	8.0	VNO, 6m Female, sex separated	AR013	AGTCAA
VNO-SF2	8.0	VNO, 6m Female, sex separated	AR014	AGTTCC
VNO-SF3	7.8	VNO, 6m Female, sex separated	AR015	ATGTCA
VNO-SM1	8.0	VNO, 6m Male, sex separated	AR016	CCGTCC
VNO-SM2	8.0	VNO, 6m Male, sex separated	AR018	GTCCGC
VNO-SM3	7.6	VNO, 6m Male, sex separated	AR019	GTGAAA
VNO-CF1	7.6	VNO, 6m Female, sex combined	AR002	CGATGT
VNO-CF2	7.6	VNO, 6m Female, sex combined	AR004	TGACCA
VNO-CF3	7.7	VNO, 6m Female, sex combined	AR005	ACAGTG
VNO-CM1	7.6	VNO, 6m Male, sex combined	AR006	GCCAAT
VNO-CM2	7.3	VNO, 6m Male, sex combined	AR007	CAGATC
VNO-CM3	7.7	VNO, 6m Male, sex combined	AR012	CTTGTA
OB-SF1	9.4	OB, 6m Female, sex separated	AR013	AGTCAA
OB-SF2	9.2	OB, 6m Female, sex separated	AR014	AGTTCC
OB-SF3	9.3	OB, 6m Female, sex separated	AR015	ATGTCA
OB-SM1	9.3	OB, 6m Male, sex separated	AR016	CCGTCC
OB-SM2	9.0	OB, 6m Male, sex separated	AR018	GTCCGC
OB-SM3	9.4	OB, 6m Male, sex separated	AR019	GTGAAA
OB-CF1	8.8	OB, 6m Female, sex combined	AR002	CGATGT
OB-CF2	9.3	OB, 6m Female, sex combined	AR004	TGACCA
OB-CF3	9.0	OB, 6m Female, sex combined	AR005	ACAGTG
OB-CM1	8.9	OB, 6m Male, sex combined	AR006	GCCAAT
OB-CM2	9.0	OB, 6m Male, sex combined	AR007	CAGATC
OB-CM3	9.3	OB, 6m Male, sex combined	AR012	CTTGTA

**Table 2:** Summary of sequencing and data and alignment statistics for each sample.

Sample ID	# input read pairs	% reads aligned	# aligned pairs	% pairs concordant	Mean insert size
MOE-SF1	17,936,897	95.4	16,756,408	89.5	174
MOE-SF2	20,343,882	95.3	19,026,235	89.5	200
MOE-SF3	19,317,876	94.3	17,828,012	87.9	180
MOE-SM1	19,292,464	90.1	16,870,239	82.9	165
MOE-SM2	17,946,239	91.6	15,997,782	84.9	166
MOE-SM3	19,684,675	92.7	17,823,615	86.7	162
MOE-CF1	15,957,907	89.8	13,790,262	82.6	164
MOE-CF2	18,146,858	93.2	16,468,092	87.2	175
MOE-CF3	18,285,912	91.2	16,091,283	83.5	171
MOE-CM1	19,360,628	95.2	18,011,836	89.5	169
MOE-CM2	17,344,326	94.7	16,073,852	89.5	175
MOE-CM3	23,414,279	77.6	16,583,279	63.6	150
VNO-SF1	19,454,769	81.2	14,691,640	65.2	156
VNO-SF2	23,880,934	88.6	20,326,802	78.2	169
VNO-SF3	23,632,351	88.8	20,223,703	79.2	166
VNO-SM1	21,999,191	85.1	17,899,156	75.0	171
VNO-SM2	16,463,769	92.5	14,846,906	85.4	177
VNO-SM3	22,234,190	90.3	19,368,457	80.6	177
VNO-CF1	20,872,570	86.6	17,298,741	76.1	167
VNO-CF2	23,265,141	94.3	21,523,031	88.5	180
VNO-CF3	20,514,901	92.5	18,528,145	85.8	165
VNO-CM1	28,682,728	87.0	23,754,542	73.0	173
VNO-CM2	22,664,866	90.8	20,026,105	83.7	169
VNO-CM3	23,963,570	90.5	20,964,674	81.4	174
OB-SF1	23,422,139	89.0	20,097,969	80.3	159
OB-SF2	20,718,443	91.1	18,381,492	84.4	164
OB-SF3	22,653,174	86.0	18,573,903	75.0	157
OB-SM1	12,631,476	86.7	10,569,714	79.0	157
OB-SM2	20,580,681	90.6	18,172,810	84.1	163
OB-SM3	22,306,877	90.4	19,600,592	83.3	162
OB-CF1	18,551,012	92.7	16,737,213	85.0	167
OB-CF2	20,766,305	92.1	18,645,696	85.3	169
OB-CF3	19,576,199	85.2	15,852,042	73.6	154
OB-CM1	21,978,571	89.3	19,014,325	81.7	160
OB-CM2	20,380,317	89.7	17,696,453	81.7	162
OB-CM3	21,601,326	91.2	19,198,089	84.9	159
